# Abnormal bihemispheric responses in schizophrenia patients following cathodal transcranial direct stimulation

**DOI:** 10.1007/s00406-012-0298-7

**Published:** 2012-02-09

**Authors:** Alkomiet Hasan, Richard Aborowa, Michael A. Nitsche, Louise Marshall, Andrea Schmitt, Oliver Gruber, Peter Falkai, Thomas Wobrock

**Affiliations:** 1 Department of Psychiatry and Psychotherapy, Georg-August University, Von-Siebold-Strasse 5, 37075 Göttingen, Germany; 2 Department of Clinical Neurophysiology, Georg-August University, Göttingen, Germany; 3 Sobell Department of Motor Neuroscience, UCL Institute of Neurology, London, UK

**Keywords:** Schizophrenia, Cortical plasticity, Cortical connectivity, Glutamate, Transcranial direct current stimulation

## Abstract

Post-mortem and in vivo studies provide evidence for a link between reduced plasticity and dysconnectivity in schizophrenia patients. It has been suggested that the association between plasticity and connectivity contributes to the pathophysiology and symptomatology of schizophrenia. However, little is known about the impact of glutamate-dependent long-term depression (LTD)-like cortical plasticity on inter-hemispheric connectivity in schizophrenia patients. The aim of the present study was to investigate LTD-like cortical plasticity following excitability-diminishing cathodal transcranial direct current stimulation (tDCS) of the left primary motor cortex (M1) and its effects on the non-stimulated right M1. Eighteen schizophrenia patients and 18 matched (age, gender, handedness, and smoking status) control subjects were investigated in this study. Corticospinal excitability changes following tDCS and intra-cortical inhibitory circuits were monitored with transcranial magnetic stimulation. On the stimulated hemisphere, cathodal tDCS increased resting motor thresholds (RMT) in both groups and decreased motor-evoked potential (MEP) sizes in healthy controls to a greater extent compared to schizophrenia patients. On the non-stimulated hemisphere, RMTs were increased and MEPs were decreased only in the healthy control group. Our results confirm previous findings of reduced LTD-like plasticity in schizophrenia patients and offer hypothetical and indirect in vivo evidence for an association between LTD-like cortical plasticity and inter-hemispheric connectivity in schizophrenia patients. Moreover, our findings highlight the impact of plasticity on connectivity. Dysfunctional *N*-methyl d-aspartate receptors or modulation of dopaminergic transmission can explain these findings. Nevertheless, the effects of antipsychotic medication still need to be considered.

## Introduction

The etiology of schizophrenia remains elusive. Neurodevelopmental alterations, as well as genetic and environmental influences, have been discussed as possible pathophysiological factors. Moreover, studies in animals and humans indicate that neural dysconnectivity, resulting from disturbed micro- and macro-circuitry, is another important neurobiological agent of the disorder [36]. It has been proposed that impairments in synaptic plasticity associated with this dysconnectivity play a central role in schizophrenia’s pathophysiology, as supported by molecular, animal and human evidence [5, 21, 38].

In contrast to long-term potentiation (LTP), there has been no detailed investigation of long-term depression (LTD) with regards to its relevance for schizophrenia. LTD is a mediator of memory storage, displays signal-to-noise-ratio regulation, and is associated with the forgetting of stored information. More specific neurobiological functions of LTD in information processing have been discussed elsewhere [18]. We have recently examined LTD-like plasticity following transcranial direct current stimulation (tDCS) in another study and found it to be abolished in schizophrenia patients compared to healthy controls [13]. We concluded in this previous publication that this specific plasticity deficit in schizophrenia might be associated with *N*-methyl d-aspartate receptors (NMDAR) dysfunction, a hyperglutamatergic state, and a reduced signal-to-noise-ratio. However, little is known about the relationship between LTD-like cortical plasticity and inter-hemispheric connectivity in schizophrenia patients. On the one hand, structural (anatomical) connectivity might be impaired but, alternatively, connectivity could be functionally defective due to impairments of synaptic plasticity in schizophrenia patients [38].

Many studies have demonstrated impaired inter-areal connectivity in schizophrenia patients. For instance, the application of both single- and double-pulse transcranial magnetic stimulation (TMS) designs has demonstrated defective facilitatory and inhibitory connections between left and right primary motor cortices and between other areas of the motor system in schizophrenia patients [4, 6, 16, 19, 34, 36]. Another approach testing inter-hemispheric connectivity involves applying a plasticity-inducing stimulation protocol to one cortical area and investigating the excitability changes at both the site of the stimulation and in more remote interconnected sites [35, 39]. Only one foregoing study has used such a setup to explore whether connectivity deficits are related to deficient plasticity of intra- and inter-hemispheric connectivities in schizophrenia patients. In this study, 1 Hz rTMS applied to the left pre-motor cortex suppressed excitability of the ipsilateral primary motor cortex (M1) in healthy controls but increased M1 excitability in schizophrenia patients [32].

In the present study, we aimed to explore the association between NMDAR-dependent non-focal LTD-like plasticity and the plasticity of inter-hemispheric connectivity in schizophrenia patients using excitability-diminishing cathodal tDCS. Therefore, the effects of cathodal tDCS on both the M1 to which it was applied and on the non-stimulated right hemisphere were tested. Evidence from studies conducted in both animals and humans suggest that cathodal tDCS offers an appropriate methodology to investigate NMDAR-dependent LTD-like plasticity in the human motor system [2, 28, 29]. Based on the theoretical framework of an association between plasticity and connectivity [38], we hypothesized that schizophrenia patients would present alterations in inter-hemispheric M1 to M1 connectivity following a plasticity-inducing stimulus. This would be expressed as a reduced response of the non-stimulated hemisphere in schizophrenia patients. Second, in this new study, we sought to replicate our prior findings of an abnormal LTD-like plasticity in schizophrenia patients in order to provide strengthen evidence for the presence of this important pathophysiological state in schizophrenia. In line with our previously published results of a deficient LTD-like cortical plasticity in schizophrenia patients, we hypothesized that schizophrenia patients would show an abnormal LTD-like plasticity in the stimulated area and that this dysfunction would be associated with alterations in inhibitory cortical networks.

## Materials and methods

### Subjects

In this experimental study, 18 schizophrenia patients from the same geographical area were recruited from inpatient and outpatient units and were compared with 18 matched healthy subjects. Subjects with a history of dermatological diseases, dementia, neurological illnesses, severe brain injuries, or brain tumors were excluded from the study. After a complete description of the study, written informed consent was obtained from each patient/healthy subject. The local ethics committee approved the protocol, and the experiments conformed to the statements of the Declaration of Helsinki.

A consensus diagnosis, according to the ICD-10 criteria for schizophrenia, was made independently by a clinical psychiatrist and a member of the study group. All subjects were right handed (laterality quotient >80) according to the Edinburgh handedness inventory [31]. Assessments of psychopathology (Positive and Negative Syndrome Scale, PANSS) [17], disease severity (clinical global impression, CGI) [12], social functioning (global assessment of functioning, GAF) [9], and duration of psychosis (DUP) were also performed in the schizophrenia patients.

All patients were treated with second-generation antipsychotics (see Table 1) and had shown a stable response to the same medication for at least 2 weeks. No patient was administered benzodiazepines, mood-stabilizers, or anticonvulsants. Six patients received additional antidepressants: duloxetine, venlafaxine, mirtazapine, escitalopram, escitalopram/mirtazapine, and venlafaxine.

**Table 1:** Antipsychotic medications received by schizophrenia patients

Patient no.	Antipsychotic medication	Dosage (mg/day)
1	Risperidone consta	50^a^
2	Aripiprazole; Quetiapine	10; 900
3	Risperidone	5
4	Olanzapine; Perazine	10; 50
5	Quetiapine	150
6	Amisulpride; Aripiprazole	200; 20
7	Aripiprazole	15
8	Quetiapine	300
9	Quetiapine; Risperidone	100; 7
10	Olanzapine	40
11	Aripiprazole	20
12	Aripiprazole; Olanzapine	15; 5
13	Risperidone; Risperidone consta	0.5; 50^a^
14	Amisulpride; Quetiapine	800; 1000
15	Flupentixol depot	0.5^b^
16	Quetiapine; Ziprasidone	400; 120
17	Aripiprazole	20
18	Quetiapine	150

Six patients received additional antidepressants: please see the Sect. “Materials and methods” for further details

^a^These patients were treated with Risperidone consta (50 mg by depot injection every 2 weeks)

^b^This patient was treated with Flupentixol depot [0.5 ml (2%) every 5 weeks]

### tDCS procedure

A commercially available DC stimulator (Eldith-Electro-Diagnostic) was used to apply a continuous current flow, through saline-soaked surface sponge electrodes (35 cm^2^). The motor-cortical electrode was placed over the representational field of the right first dorsal interosseus muscle (FDI) as identified by TMS, and the other electrode was located contralaterally above the right orbit. To produce long-lasting excitability changes (up to 1 h), a constant current was applied with an intensity of 1 mA for 9 min [27].

### TMS procedure

According to previous publications [13], subjects were seated in a comfortable reclining chair with their arms supported passively. Electromyographic (EMG) recordings from the right and left FDI were made using surface electrodes. Raw signals were amplified, bandpass-filtered (2 Hz–10 kHz), and digitized using a commercially available amplifier (Keypoint, Medtronic, Denmark). Each EMG recording was manually analyzed off-line.

Transcranial magnetic stimulation was performed over the left and right motor cortex with a standard 70 mm-TMS figure-of-eight magnetic coil using a biphasic MagPro-X-100 magnetic stimulator (Medtronic). The optimal coil position was defined as the stimulation site that produced the largest motor-evoked potential (MEP) at moderately suprathreshold stimulation intensities in the resting right and left FDI muscles. The optimal position was marked to ensure that the coil was held in the correct position throughout. The coil was held tangentially to head with the handle pointing backwards and at an angle of 45° lateral to the midline to induce a posterior–anterior-directed current flow within the cortex.

### Experimental design to monitor and characterize excitability changes

To reduce the duration of each experimental session, thereby minimizing distress for the schizophrenia patients, we focused on measuring the resting motor threshold [resting motor thresholds (RMT), expressed as percentage maximum stimulator output (%MSO)], the TMS intensity that produced MEPs averaging 0.7–1.3 mV (S1 mV, expressed as %MSO), single-pulse MEP amplitudes at an intensity of S1 mV, and the contralateral cortical silent period (CSP). All parameters were recorded for the left and right hemispheres before and after tDCS of the left hemisphere. The RMT, expressed as a percentage of the maximum stimulator output, was defined as the lowest intensity that produced an MEP >50 μV in the relaxed FDI in at least five out of ten trials [13, 43]. To monitor the effects of tDCS on motor cortex excitability and plasticity, 40 TMS-elicited MEPs were recorded from the motor-cortical representation of the right and left FDI muscles before and after tDCS. TMS intensity was adjusted to evoke MEPs of 1 mV on average for baseline determination and was kept unchanged for the after-effect assessment.

Cortical silent period was measured in the moderately tonically active FDI (25–30% of maximal contraction) by stimulating the contralateral motor cortex (left or right) with TMS intensities of S1 mV. Ten trials were performed before and after tDCS for each hemisphere and the mean CSP duration calculated. CSP duration was defined as the latency from MEP onset to the return of any voluntary EMG activity [3].

As the aim of the study was to examine plasticity disturbances and their mechanisms in schizophrenia, MEPs were recorded before tDCS and 5 min after stimulation over the left and right M1. Foregoing studies had indicated that these time points are promising for obtaining an LTD-like plasticity effect. All other measures were recorded in the same order (first left hemisphere then right hemisphere), at baseline and within 30 min after stimulation [27]. S1 mV was only adjusted for the assessment of CSP after tDCS.

### Statistics

SPSS 18 for Windows was used for all statistical analyses. Level of significance was set at α = 0.05. For gender, hand preference, and smoking status, χ²-tests were computed to test for a different distribution between groups. An independent *t* test was used to compare mean ages between the groups. MEP size was calculated as the mean MEP amplitude individually and then inter-individually before and after stimulation.

Separate repeated measures ANOVAs (RM-ANOVA) were calculated with the following dependent variables: single-pulse MEP amplitude, RMT, S1 mV, and CSP. “Group” (healthy controls vs. schizophrenia patients) served as the between-subject factor, and “time” (baseline vs. post-stimulation) and “hemisphere” (left vs. right) as the within-subject factors. This three-way ANOVA was based on the assumption of time-specific and hemispheric-specific effects of tDCS.

To determine more specifically whether the MEP amplitudes before and after tDCS differed within and between groups, Student’s *t* tests (independent-samples for the inter-group comparisons, and paired-samples for the intra-group pre- vs. post-comparisons, two-tailed, *p* < 0.05) were performed where appropriate (interaction in the ANOVA model). Pearson correlations between post- or pre-ratios of the dependent variables on both hemispheres were performed to test for an inter-hemispheric association of the stimulation effects.

Pearson correlations between dependent variables and PANSS values, chlorpromazine (CPZ) equivalents, GAF, CGI and duration of psychosis were performed in the patient group. Sphericity was tested with the Mauchly’s test and, if necessary (Mauchly’s test <0.05), the Greenhouse–Geisser correction was applied. Data are presented as mean ± SD unless otherwise indicated. The results of this exploratory study are presented without error probability corrections.

## Results

### Sociodemographic and clinical characteristics

Groups were matched according to age (*p* = 0.429), gender (*p* = 0.717), smoking status (*p* = 0.317) and handedness (*p* = 1.000). Table 2 supplies a detailed review of the sociodemograhic characteristics and clinical scores. Schizophrenia patients received moderate dosages of antipsychotics, as expressed by CPZ equivalents (444.28 ± 388.64).

**Table 2:** Demographic and clinical characteristics of the subjects

Variable	Healthy controls	Schizophrenia patients
*N*	18	18
Gender	12 M, 6 F	13 M, 5 F
Age (years)	31.50 ± 9.94	34.33 ± 11.24
Right handed	18	18
Smoker	7	10
PANSS score		
Total	–	58.94 ± 14.18
Positive	–	13.28 ± 4.51
Negative	–	16.56 ± 4.10
General	–	29.11 ± 7.70
GAF	–	54.72 ± 11.42
CGI	–	4.67 ± 0.94
CPZ (daily)	–	444.28 ± 388.64
Duration of psychosis (years)	–	4.391 ± 3.32

Data are presented as mean ± SD

*PANSS* Positive and Negative Syndrome Scale, *GAF* global assessment of functioning, *CGI* clinical global impression, CPZ chlorpromazine equivalent dose

### Results of RM-ANOVAs (factors “time,” “hemisphere” and “group”)

#### MEP amplitudes (see Fig. 1; Table 3)

For MEP amplitudes, RM-ANOVA revealed significant effect of “time” and “hemisphere”. The interactions “time × group” and “time × hemisphere” were significant, but RM-ANOVA revealed no significance for the “hemisphere × group” and the “time × hemisphere × group” interactions.

**Fig. 1:**
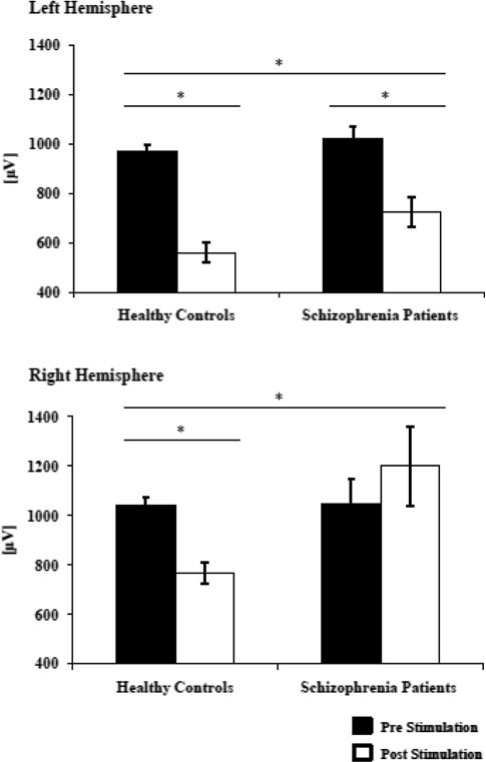
Absolute change of MEP size pre- and post-tDCS stimulation in healthy controls and schizophrenia patients. Baselines did not differ between groups on the left hemisphere (*p* > 0.05) and on the right hemisphere (*p* > 0.05). Cathodal tDCS reduced the MEP sizes on the stimulated left hemisphere in healthy controls (*p* < 0.001) and in the schizophrenia group (*p* = 0.001), whereas the decrease in MEP size was greater in the control group (*p* = 0.049). On the non-stimulated hemisphere, cathodal tDCS reduced MEP size in the control group (*p* = 0.026), but not in the schizophrenia group (*p* = 0.376). Therefore, MEP sizes were smaller in the control group (*p* = 0.031). *Short horizontal line*: significant differences before and after stimulation (paired-sample *t* test). *Long horizontal line*: significant “time × group” interaction (ANOVA)

**Table 3:** Results of the RM-ANOVAs with regard to different parameters

	*df*, error	*F* value	*p* value
MEP amplitudes			
Time	1, 34	13.99	0.001*
Hemisphere	1, 34	11.64	0.002*
Time × group	1, 34	5.95	0.020*
Time × hemisphere	1, 34	7.06	0.012*
Hemisphere × group	1, 34	0.903	0.349
Time × hemisphere × group	1, 34	2.012	0.165
S1 mV			
Time	1, 34	33.20	<0.001*
Hemisphere	1, 34	15.13	<0.001*
Time × group	1, 34	5.31	0.027*
Time × hemisphere	1, 34	7.48	0.010*
Hemisphere × group	1, 34	0.571	0.455
Time × hemisphere × group	1, 34	2.913	0.213
RMT			
Time	1, 34	24.45	<0.001*
Hemisphere	1, 34	5.88	0.021*
Time × group	1, 34	3.34	0.076
Time × hemisphere	1, 34	10.07	0.003*
Hemisphere × group	1, 34	0.112	0.740
Time × hemisphere × group	1, 34	0.643	0.446
CSP			
Time	1, 34	8.07	0.008*
Hemisphere	1, 34	0.235	0.631
Time × group	1, 34	1.04	0.315
Time × hemisphere	1, 34	2.18	0.150
Hemisphere × group	1, 34	0.79	0.381
Time × hemisphere × group	1, 34	1.24	0.273

These analyses show significant “time × group” and “time × hemisphere” interactions for MEP-variables (details are provided in the Sect. “Results”). These findings are confirmed by the results of the RM-ANOVA for S1 mV. In contrast to our predictions, analyses did not reveal a significant “time × group” interaction for CSP

*ANOVA* analysis of variance

* *p* < 0.05

For the left hemisphere, paired *t* tests showed a significant reduction in MEP amplitudes after cathodal tDCS in healthy controls (*t* = 7.691, *df* = 17, *p* < 0.001) and in schizophrenia patients (*t* = 4.028, *df* = 17, *p* = 0.001). For the right hemisphere, paired *t* tests showed a reduction in MEP amplitudes after cathodal tDCS in healthy controls (*t* = 2.436, *df* = 17, *p* = 0.026), but not in schizophrenia patients (*t* = −0.909, *df* = 17, *p* = 0.376). In both groups, no difference in MEP sizes (comparing left vs. right hemisphere) before stimulation could be detected (healthy controls: *t* = 1.731, *df* = 17, *p* = 0.102; schizophrenia patients: *t* = 0.453, *df* = 17, *p* = 0.657). However, after stimulation, healthy controls had no difference in the MEP sizes between hemisphere (*t* = 1.731, *df* = 17, *p* = 0.102), whereas schizophrenia patients displayed higher MEP values on the right hemisphere (*t* = 2.654, *df* = 17, *p* = 0.017).

Independent-samples *t* tests did not reveal a significant between-group difference of 1 mV-MEPs at baseline on the left (*t* = 1.02, *df* = 34, *p* = 0.313) or right hemispheres (*t* = 0.09, *df* = 34, *p* = 0.927), but there was found to be a significant difference after stimulation between groups on the left (*t* = 2.041, *df* = 34, *p* = 0.049) and right hemispheres (*t* = 2.244, *df* = 34, *p* = 0.031).

#### RMT (see Tables 3/4)

Results of the RM-ANOVAs (within-group comparisons and between-group comparisons) are presented in Tables 3 and 4. In the comparison of the left and right hemispheres, healthy controls and schizophrenia patients had significantly higher RMT values after stimulation on the left hemisphere compared to the right hemisphere (*p* = 0.048; *p* = 0.024).

**Table 4:** Values and statistical results of RMT and S1 mV

	Healthy controls	Schizophrenia patients	Statistics between groups
S1 Mv left hemisphere (%)			
Pre-tDCS	53.78 ± 10.55	59.44 ± 10.86	*p* = 0.132
Post-tDCS	56.33 ± 11.04	61.5 ± 10.35	*p* = 0.168
Statistics within group	*p* < 0.001*	*p* < 0.001*	
RMT left hemisphere (%)			
Pre-tDCS	44.61 ± 7.57	49.44 ± 9.84	*p* = 0.118
Post-tDCS	46.78 ± 8.46	51.61 ± 9.73	*p* = 0.132
Statistics within group	*p* < 0.001*	*p* < 0.001*	
CSP left hemisphere (ms)			
Pre-tDCS	137.23 ± 34.30	156.32 ± 41.62	n.t.
Post-tDCS	139.22 ± 41.11	159.40 ± 39.93	n.t.
Statistics within group	n.t.	n.t.	
S1 mV right hemisphere (%)			
Pre-tDCS	48.06 ± 7.10	56.39 ± 10.30	*p* = 0.010*
Post-tDCS	49.78 ± 7.31	56.17 ± 9.65	*p* = 0.037*
Statistics within group	*p* < 0.001*	*p* = 0.795	
RMT right hemisphere (%)			
tDCS	42.06 ± 5.73	48.56 ± 8.85	*p* = 0.016*
Post-tDCS	43.39 ± 5.95	48.00 ± 8.84	*p* = 0.072
Statistics within group	*p* < 0.001*	*p* = 0.523	
CSP right hemisphere (ms)			
Pre-tDCS	137.08 ± 31.52	153.58 ± 47.05	n.t.
Post-tDCS	151.92 ± 31.45	158.45 ± 47.11	n.t.
Statistics within group	n.t.	n.t.	

Statistics between groups are independent-samples *t* tests pre- or post-tDCS. Statistics within a group are paired-samples *t* tests pre- vs. post-tDCS. Data are presented as mean ± SD

*RMT* resting motor thresholds, *S1* mV intensity to evoke MEP of 1 mV, *CSP* contralateral silent period, *n.t.* not tested, due a lacking interaction in the ANOVA (see “Statistics” and “Results” section)

* *p* < 0.05

### CSP (see Tables 3/4)

For CSP, RM-ANOVA revealed only a significant effect of time [*F* (1, 34) = 8.027, *p* = 0.008] but no further significant main effects or interactions. Therefore, no further post hoc analyses were conducted.

#### S1 mV (see Tables 3/4)

Results of the RM-ANOVAs [within-group comparisons (post vs. pre) and between-group comparisons] are presented in Tables 3 and 4. As expected, these results confirm the findings from the MEP amplitudes.

### Pearson correlations

Analyses did not reveal any significant correlation of post-/pre-ratios between left and right hemispheres in either group, indicating independent neuroplastic alterations in both hemispheres.

### Influence of clinical variables and medication on TMS parameters

Pearson correlation analysis revealed a significant correlation between only antipsychotic medication, expressed as CPZ equivalents, and the left hemispheric RMTs before (*p* = 0.023) and after (*p* = 0.031) stimulation. The correlations between the main outcome parameters, the clinical variables PANSS, CGI, GAF, and duration of psychosis were not significant.

To control for possible effects of antidepressant medication, a RM-ANOVA with the factors “group” (medication/no medication), “time,” and “hemisphere” was conducted within the patient group. This RM-ANOVA displayed no significant “time × group,” “hemisphere × group,” or “group × time × hemisphere” interactions, but, as expected, a main effect of “hemisphere” (*p* = 0.012), and “time × hemisphere” interaction (*p* = 0.016).

## Discussion

The results of the present tDCS study provide indirect evidence for an impaired LTD-like plasticity of inter-hemispheric connectivity and also replicate our prior finding of a reduced non-focal LTD-like plasticity in the left primary motor cortex of schizophrenia patients. tDCS to the left M1 reduced MEPs in healthy subjects to a greater extent than in schizophrenia patients. In the non-stimulated right hemisphere, MEPs were only reduced in healthy controls, whereas schizophrenia patients showed an abolished LTD-like plasticity response following tDCS to the left hemisphere.

Schizophrenia patients had higher RMT values on the right hemisphere compared to healthy controls and tDCS failed to modulate RMT in the right hemisphere in the patient group.

### Effects on the stimulated left hemisphere

One previously published study has shown abolished LTD-like plasticity following tDCS to the stimulated hemisphere in schizophrenia patients [13]. This was linked to dysfunctional glutamatergic NMDAR-dependent neurotransmission in patients, although it was noted that a possible confounding effect of antipsychotic medication on dopaminergic transmission should be considered, as should the difficulty of translating findings from molecular to human models. However, we speculated, based on animal and molecular studies, that a general hyperglutamatergic state with a subsequent increase in glutamatergic transmission and an amplification of intracellular calcium [23] could abolish the development of LTD following tDCS [13].

In this present, independently conducted study in which we examined a group of partially newly recruited patients and healthy controls, we found a reduced LTD-like plasticity in schizophrenia patients, which is in line with our prior results of dysfunctional LTD-like plasticity. The possible molecular (dysfunctional NMDARs) and functional (modulation of signal-to-noise-ratio) mechanisms underlying these plasticity deficits following cathodal tDCS have been discussed extensively elsewhere [13], but a hyperglutamatergic state is within the realms of possibility. It should be noted that MEPs in the left hemisphere were smaller after stimulation compared to the right hemisphere in schizophrenia patients. This shows that, in this study, there was a reduced response by the stimulated hemisphere, while any plasticity response by the non-stimulated hemisphere was abolished. This highlights the link between plasticity and connectivity, and the complex interplay between hemispheres is discussed in detail below. In the present study, we found reduced MEP amplitudes as well as enhanced RMT. Although the enhanced RMT after cathodal tDCS in our study sample is in line with other publications [1], it has not been described in all publications [30]. The reasons for these different results with regard to RMT are unclear at present. In summary, our recent results provide additional evidence for a reduced plasticity response in the stimulated hemisphere of schizophrenia patients following cathodal tDCS.

### Effects on the non-stimulated right hemisphere

Motor-evoked potentials in the non-stimulated hemisphere were only suppressed, and RMT enhanced, in the control group, indicating that the plasticity response in the stimulated left M1 might have an impact on the excitability of the interconnected non-stimulated right M1 in healthy controls, but not in schizophrenia patients. Therefore, our results are indicative of a disturbed plasticity of the inter-hemispheric M1-to-M1 connectivity in schizophrenia patients. There is little knowledge about the cross-modulation of excitability following tDCS, but animal and human studies have discussed the possibility that motor-cortical representations of the digits are interconnected between both motor cortices [41]. Aberrant anatomical wiring of both motor cortices, alterations in subcortical pathways (e.g., basal ganglia) [8, 10], and white matter changes [7] could cause connectivity deficits and might underlie our findings.

Our findings in healthy subjects contrast those of Lang and colleagues, who showed in one report that cathodal tDCS-induced MEP suppression is limited to the stimulated M1 in healthy subjects [22]. However, the sample size of this previous report was relatively low (*n* = 8) and might therefore have been underpowered to detect contralateral excitability alterations. The results of the present study are in principle accordance with numerous rTMS studies, in which it has been shown that stimulation of the motor cortex of one hemisphere induces facilitatory or inhibitory effects of the contralateral motor cortex [33, 39, 41]. Therefore, it is likely that stimulation of the left M1 causes excitability changes in the right M1. However, the directions of these changes are variable and may depend on different, as yet unidentified, factors [37].

### From plasticity to connectivity

Our finding of a reduced LTD-like plasticity in the stimulated M1 and an abolished LTD-like plasticity in the non-stimulated M1 indicates an indirect link between reduced cortical plasticity and dysconnectivity in schizophrenia patients. This extends the findings of Oxley et al. [32], who showed that inhibitory 1-Hz rTMS applied to the pre-motor areas fails to modulate excitability of the primary motor cortex. The neurobiological mechanisms of transcranial brain stimulation techniques are not fully understood, but animal and human studies indicate that tDCS modulates cortical plasticity via NMDARs [2, 11, 29]. Furthermore, a causal relationship between dysfunctional NMDARs, plasticity alterations, and neural dysconnectivity in schizophrenia has been discussed [38]. Our recent findings of a reduced inter-hemispheric response following cathodal tDCS in schizophrenia patients may reflect plasticity-dependent connectivity impairments in schizophrenia patients and may be linked to abnormal activation of various neurotransmitters (e.g., glutamate, GABA) and/or neuromodulatory (e.g., dopamine, acetylcholine) systems [36, 38]. However, our experimental setup cannot be used to answer this question in detail because we tested connectivity with only an indirect approach.

The proposed intra- and inter-hemispheric dysconnectivites in schizophrenia are supported by several lines of evidence, as recently reviewed by our study group [36]. Post-mortem studies have revealed a loss of oligodendrocytes and reduced expression of oligodendrocyte-related genes, leading to subsequent disturbance of microconnectivity in schizophrenia patients [14, 15, 36]. Diffusion tensor imaging studies have shown depleted myelin membranes and decreased white matter anisotropy in different parts of the cortex of schizophrenia patients [8, 20], while neurophysiological studies with TMS have displayed deficient inhibitory and facilitatory pathways within the motor system [6, 16, 19, 34]. Finally, EEG and magnetoencephalography studies have reported impaired neural oscillations and reduced phase synchronization as a marker for functional dysconnectivity in schizophrenia patients [40]. Not only are our results in line with these studies, they add new insight by showing that an abnormal response of NMDAR-dependent non-focal LTD-like plasticity has an impact on inter-hemispheric connectivity in schizophrenia patients.

### Cortical silent period

We previously reported that an association between abolished cortical LTD-like plasticity and intra-cortical inhibition (CSP) [13], in addition to alterations to various neurotransmitter systems, reflected by cortical excitability changes, are a common finding in schizophrenia patients [24]. In contrast to our predictions, we were not able to find a statistically different CSP between groups, even though schizophrenia patients had a trend wise prolonged CSP at baseline (left hemisphere: 156 vs. 137 ms; right hemisphere 153 vs. 137 ms), in accordance with previous publications of our study group [42]. However, it should be noted that differences in disease states and medication could lead to conflicting results with regard to CSP [24, 42].

### Limitations

Some important limitations should be considered in this study. First, we cannot rule out an impact of antipsychotic medication on our results. We did not find a correlation between CPZ equivalents and our dependent variables but, nevertheless, dopaminergic modulation has been shown to have prominent effects on cortical excitability and plasticity. Furthermore, it has to be taken into consideration that dopaminergic activation and de-activation, as well as NMDAR modulations, could, at least partially, explain our findings [25, 26]. The effect of dopamine on cortical and subcortical excitability and plasticity is very complicated, and long-term treatment with antipsychotic drugs makes the associations even more complicated. Therefore, the effects of dopamine must remain in the back of one’s mind when interpreting our present findings and, moreover, these findings should be replicated in a larger and unmedicated sample of patients.

Second, we used an indirect approach to test inter-hemispheric connectivity in this study. Therefore, we cannot verify a specific link between reduced plasticity and connectivity, but rather an association between the two. In future, alternative setups with specific measures of connectivity (e.g., combination of tDCS with EEG/MEG or diffuser tensor imaging) should be used to provide more information about the link between plasticity and connectivity.

Finally, our results would not survive correction for multiple testing. An adjustment for multiple testing would decrease the test power of this exploratory study. This would result in a situation in which the probability of finding existing mean differences would be very low. Therefore, studies with larger samples are needed to confirm a causal relationship for these findings.

### Conclusions

In summary, this tDCS study shows that patients with schizophrenia display reduced LTD-like plasticity following tDCS at the site of stimulation and abolished LTD-like plasticity on the non-stimulated side when compared to healthy controls. Our findings add some knowledge to the current discussion concerning plasticity- and NMDAR-dependent connectivity deficits in schizophrenia patients. However, different confounding factors, such as antipsychotic medication and alternative mode of actions (dopamine transmission, GABAergic transmission), are likely to have some involvement and cannot be ruled out as potential sources of modulation. The novel finding of an impaired plasticity-dependent inter-hemispheric connectivity is a new piece that may help to complete the jigsaw of the interaction between plasticity and dysconnectivity in schizophrenia.
